# Lung disease recognition methods using audio-based analysis with machine learning

**DOI:** 10.1016/j.heliyon.2024.e26218

**Published:** 2024-02-17

**Authors:** Ahmad H. Sabry, Omar I. Dallal Bashi, N.H. Nik Ali, Yasir Mahmood Al Kubaisi

**Affiliations:** a Department of Medical Instrumentation Engineering Techniques, Shatt Al-Arab University College, Basra, Iraq; b Medical Technical Institute, Northern Technical University, 95G2+P34, Mosul, 41002, Iraq; c School of Electrical Engineering, College of Engineering, Universiti Teknologi MARA, 40450 Shah Alam, Selangor, Malaysia; d Department of Sustainability Management, Dubai Academic Health Corporation, Dubai, 4545, United Arab Emirates

**Keywords:** Lung disease recognition, Audio-based analysis, Machine learning, Lung sounds, Respiratory sounds, Audio processing, Feature extraction, Classification

## Abstract

The use of computer-based automated approaches and improvements in lung sound recording techniques have made lung sound-based diagnostics even better and devoid of subjectivity errors. Using a computer to evaluate lung sound features more thoroughly with the use of analyzing changes in lung sound behavior, recording measurements, suppressing the presence of noise contaminations, and graphical representations are all made possible by computer-based lung sound analysis. This paper starts with a discussion of the need for this research area, providing an overview of the field and the motivations behind it. Following that, it details the survey methodology used in this work. It presents a discussion on the elements of sound-based lung disease classification using machine learning algorithms. This includes commonly prior considered datasets, feature extraction techniques, pre-processing methods, artifact removal methods, lung-heart sound separation, deep learning algorithms, and wavelet transform of lung audio signals. The study introduces studies that review lung screening including a summary table of these references and discusses the literature gaps in the existing studies. It is concluded that the use of sound-based machine learning in the classification of respiratory diseases has promising results. While we believe this material will prove valuable to physicians and researchers exploring sound-signal-based machine learning, large-scale investigations remain essential to solidify the findings and foster wider adoption within the medical community.

## 1 Introduction

### 1.1 The necessity of this study

World Health Organization (WHO) epidemiological statistics on respiratory disorders indicate that there are 30 million people with asthma and 210 million people with chronic obstructive pulmonary disease (COPD) worldwide. According to studies, 15 million and 25 million people in India suffer from asthma [[1], [2], [3]]. In order to evaluate the condition of the cardiopulmonary system, doctors frequently employ the non-invasive and affordable lung auscultatory approach [4,5]. Lung sounds are the noises made when the lungs vibrate as air passes through them during the respiratory process [6]. It truly offers information about how the lungs work and so is essential in identifying lung illnesses. Lung sounds are often divided into two categories: aberrant or adventitious lung sounds. The typical lung sounds consist of the Vesicular, Bronchial, Broncho-Vesicular, and Tracheal. Depending on how long it lasts, the adventitious lung sounds can either be continuous or discontinuous. Detecting respiratory infections early and monitoring them carefully are crucial for successful treatment. Lung auscultation, which involves using a stethoscope to listen to a patient's lung sounds, is a common clinical practice for diagnosing breathing problems. These sounds are typically categorized as either normal or abnormal. Crackles, wheezes, and squawks are the most common abnormal sounds heard above normal lung sounds, and their presence often indicates a lung condition [[7], [8], [9]].

Normal lung sound typically follows cyclic patterns that depict the respiratory process' air passage. Chronic lung airflow blockage that interferes with regular breathing and is not completely reversible is a feature of pulmonary illnesses [10]. Despite providing relatively direct and objective information, auscultation with a stethoscope is only a qualitative diagnostic tool [11,12]. However, due to a variety of factors, including inter and intra-observer variability, subjectivity errors in differentiating fine sound patterns, frequency attenuation, etc., auscultation diagnosis findings are frequently subpar [13]. The stethoscope does not provide a frequency-independent sound transmission since it non-linearly increases sounds below 112 Hz [14] and suppresses sounds over 120 Hz [15]. It has no mechanism of recording, no quantitative description, and poor sensitivity. In order to distinguish between normal and atypical sounds when using a stethoscope to diagnose a disease, a doctor must have the necessary training and experience. This skill also depends on the clinician's expertise and hearing abilities. Previous scholars have quantitatively demonstrated the effect of observer variability.

As mentioned in the study [16], two observers missed the presence of accidental sounds 24% of the time. According to Fletcher [17], there are several different ways that doctors might detect emphysema using stethoscopes. According to Elphick et al. [18], 28% of the time, doctors make the wrong diagnosis as a result of their failure to appropriately recognize respiratory symptoms in adults with auscultation. Another crucial factor that contributed to the incorrect diagnosis is the auscultatory examination of LS's poor signal-to-noise ratio, which revealed that “Thoracic lung sounds have relatively low amplitude compared with a background noise of heart and muscle sounds" [19]. As a result, the LS used for analysis is frequently damaged and degraded. Electronic auscultation was developed as a result of further study, and it has the advantages of signal amplification and ambient noise reduction, resulting in an improved signal-to-noise ratio [20]. The inability of the human ear to distinguish between closely spaced acoustic adventitious frequencies and misinterpretation, however, continue to be major obstacles in lung sound analysis [21].

The use of computer-based automated approaches and improvements in lung sound recording techniques have made lung sound-based diagnostics even better and devoid of subjectivity errors. Using a computer to evaluate lung sound features more thoroughly with the use of analyzing changes in lung sound behavior, recording measurements, suppressing the presence of noise contaminations, and graphical representations are all made possible by computer-based lung sound analysis [22].

Therefore, the advances in computer-based diagnosis such as machine and deep learning algorithms offer an enormous advantage of the removal of the subjectivity of auscultation of lung sound, analysis, and storage. Therefore, it offers a measurable, objective, and highly accurate outcome [[23], [24], [25], [26]]. Each result of such an automated study can be saved for follow-up and examination in the future [27]. Because of their heavily overlapping feature distributions, lung sounds are challenging tasks to classify [28]. Large datasets of lung sounds and spectrograms can be used to train deep neural networks without prior knowledge of specific lesions. This enables accurate detection of lung disease status with high sensitivity and specificity, even without relying on pre-established lesion-based criteria. The primary advantages of this automated disease identification method include consistent model predictions, high specificity, and efficient results generation. Consequently, finding efficient machine learning with feature extractions is crucial for accurate categorization, which this study attempts to achieve by reviewing element techniques of a machine and deep learning models toward lung sound classification.

The explosive growth of data has rendered traditional analysis methods inadequate due to time limitations and the requirement for specialized medical knowledge. In response, researchers have explored various AI-powered strategies to automate respiratory sound signal categorization. These include machine learning (ML) techniques like hidden Markov models (HMMs) and support vector machines (SVMs) [9], as well as deep learning (DL) architectures like convolutional neural networks (CNNs), residual networks (ResNets) [29], long short-term memory (LSTM) networks [30], and recurrent neural networks (RNNs) [31].

This research explores how existing deep learning architectures and models perform in the context of classifying lung sound signals as either abnormal or healthy. This review aims to provide a clear understanding of how Deep Learning networks can analyze recorded acoustic data to extract meaningful features of lung disease, reduce data complexity, address data imbalance issues, and ultimately improve prediction accuracy.

The rest of this work is organized as follows: Subsection 1.2 introduces an overview and study motivations followed by the contribution and review structure. Section 2 presents the survey methodology, while Section 3 presents a discussion of the elements of sound-based lung disease using machine learning algorithms. This includes subsections of the commonly considered dataset in the literature, feature extraction of lung sound signals, Pre-processing of lung sound signals, artifact removal methods, lung-heart sound separation, deep learning algorithms, and wavelet transform. Section 4 introduces studies that review lung screening including a summary table of these references. Section 5 discusses the literature gaps in the existing studies. Finally, in Section 6, the study's conclusions are described.

### 1.2 An overview and motivations

Lung diseases are a major global health concern, affecting millions of people worldwide. Early diagnosis and intervention are crucial for successful treatment and improving patient outcomes. Traditional lung disease diagnosis relies on methods like chest X-rays, CT scans, and lung function tests. However, these methods can be expensive, time-consuming, and invasive. Despite its potential, the field of audio-based lung disease recognition using machine learning is still in its early stages of development. A comprehensive review paper is needed to.

• Summarize the state-of-the-art: Provide an overview of existing research, including different techniques, algorithms, and datasets used.
• Identify challenges and limitations: Highlight areas that require further research and development.
• Provide future research directions: Suggest potential avenues for advancement and innovation.
• Promote awareness and adoption: Encourage wider use and acceptance of this promising technology in clinical practice.

Therefore, by conducting a comprehensive review of audio-based lung disease recognition using machine learning, this paper aims to contribute to the advancement of this field and ultimately lead to more efficient and accurate diagnosis of lung diseases, improving patient outcomes and reducing the burden on healthcare systems.

Precisely classifying medical images is vital for patient care and education [32,33]. Existing methods have limited performance and demand substantial time and effort for feature analysis and selection. Deep neural networks, especially CNNs, have demonstrated promising results in various image classification tasks. The lack of readily available medical image databases hinders progress due to the expertise needed for categorization. This paper aims to provide a comprehensive, beginner-friendly review of deep learning applications in diagnosing medical conditions. This review moderately contributes to existing knowledge by offering a concise and obvious introduction to deep learning in medical diagnosis. Three key questions guide this work.

• How diverse are deep learning's applications in diagnosing medical conditions?
• Can deep learning completely replace doctors?
• Does deep learning have long-term viability, or will it be superseded by other technologies?

### 1.3 Study contributions

This paper makes several important contributions to the field of lung disease diagnosis using audio-based analysis with machine learning.

1. This paper comprehensively analyzes the theoretical foundations and practical applications of deep learning in medical lung diagnosis. This comprehensive overview benefits researchers and practitioners by summarizing the state-of-the-art in this rapidly evolving field.
2. The paper defines key terms like “breath," “respiratory," “lung sounds," “extrinsic allergic alveolitis," and “pulmonary." This ensures clarity and understanding for a broader audience.
3. A categorization system for lung disease diagnosis methods is presented, highlighting the use of auscultation systems. This provides a structured overview of various diagnostic approaches and emphasizes the importance of auscultation in this context.
4. The paper introduces a respiratory system sound diagnosis framework, offering a common understanding of the inquiry and process of diagnosing respiratory problems. This framework serves as a valuable reference for researchers and practitioners working in this area.
5. This research presents a valuable contribution through its in-depth analysis of current studies exploring data augmentation techniques for background respiratory sounds. This analysis provides valuable insights for improving the accuracy and robustness of lung sound-based diagnosis.
6. The review underscores the importance of integrating deep learning convolutional neural networks (CNNs) into lung auscultation screening. This emphasizes the potential of deep learning to enhance the accuracy and efficiency of lung disease diagnosis.
7. The paper concludes by offering numerous recommendations for future research opportunities. This paves the way for further advancements in the field of lung disease diagnosis using audio-based analysis with machine learning.

## 2 Survey methodology

**Fig. 1:**
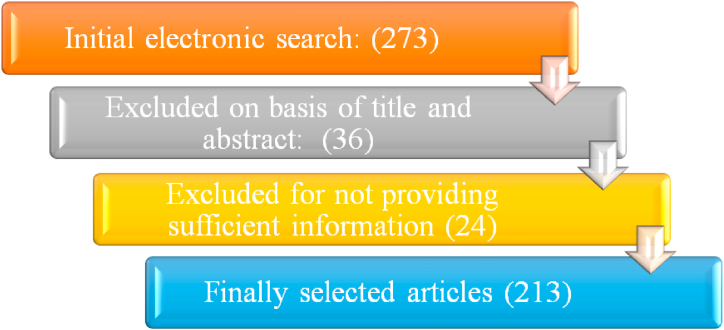
Flow chart of reference selection criteria.

When developing our publication selection criteria, the design of the explore study, and the standards for data extraction, we followed the guidelines [34] that address the systematic literature review. Additionally, we employed an efficient procedure and straightforward in line with the methodological strategy suggested in Ref. [35]. In the same context, a preliminary research strategy was presented that includes the research objective and various keyword combinations and information. This article considers “audio" to encompass sound, voice, and speech, all three of which are relevant to the respiratory system disease and lung diagnosis. The selection criteria for references included in this study are visualized in Fig. 1.

**Fig. 2:**
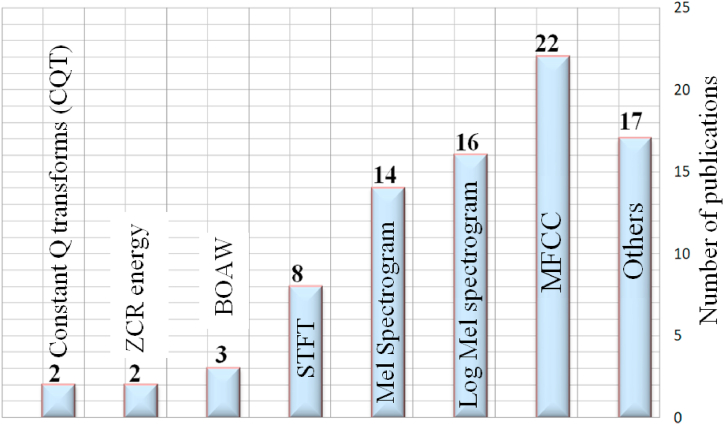
Statistics of feature extraction techniques in sound-based classification.

Among the papers we examined, 9.4% used the STFT approach for feature extraction. Other popular methods included Mel spectrograms (16.4%), log-mel spectrograms (18.5%), and MFCCs (25.5%). The remaining 30% explored various other methods like ZCR energy, CQT, and a bag of words (see Fig. 2). Our review revealed that feature extraction methods are crucial for sound classification in lung and respiratory disease diagnosis. We found that STFT was used in 9.4% of the papers, followed by Mel spectrograms (16.4%), log-mel spectrograms (18.5%), and MFCCs (25.5%). The remaining papers explored other methods like ZCR energy, CQT, and a bag of words (Fig. 2).

In the sections that follow, we use additional statistical representations that allow us to see the relationships between various analysis elements along various axes in order to do a more in-depth analysis. It enables locating the research holes in the current body of literature. In the following section, the methodologies utilized in the evaluated studies are covered.

## 3 Discussion of the system elements

**Fig. 3:**
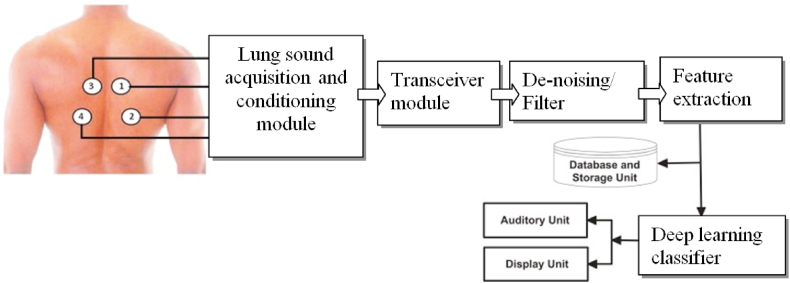
Sound-based lung disease classifier based on Deep Learning networks.

This paper discusses the system's main elements of the existing methods that are used to categorize lung sounds by deep learning algorithms such as [26,[36], [37], [38]]. Therefore, it is possible to design a setup diagram for the sound-based lung disease classifier based on Deep Learning networks as depicted in Fig. 3.

Since sound recordings are prone to noise, studies have improved data-cleaning techniques. These techniques have a significant impact on whether learning models perform better or worse. Additionally, a fascinating indication of feature extraction approaches was provided, and the distinctiveness of the currently used feature extraction methods was discussed. We focused on the associated techniques used for feature extractions as well as sound/audio datasets in order to broaden the scope of this literature review. Finally, we consider the advantages and disadvantages of the classifiers with regard to their overall performance in this section by analyzing the various classification techniques described in earlier studies.

**Fig. 4:**
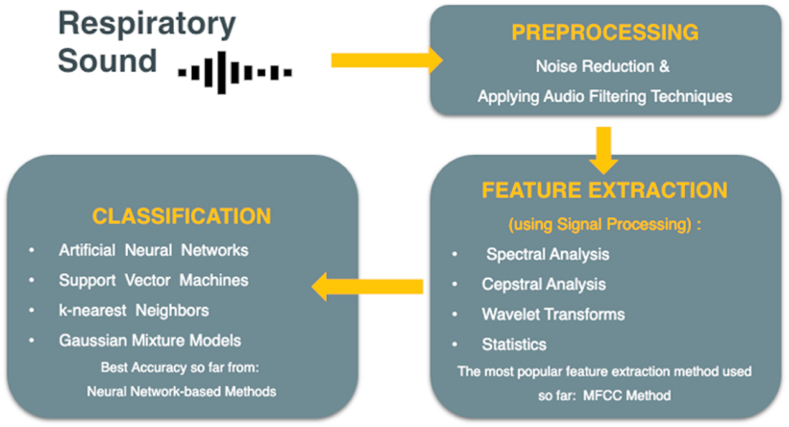
**Workflow from preprocessing to classification** [62].

Most existing projects for lung disease diagnosis using respiratory sounds follow a three-stage machine learning pipeline: 1) the preprocessing, which involves removing unwanted noise and preparing the sound data for further analysis using audio filtering and noise-reduction techniques, 2) the feature extraction, which involves extracting relevant characteristics from the preprocessed sound data using signal processing methods like spectral analysis [[39], [40], [41], [42]], cepstral analysis [[43], [44], [45]], wavelet transforms [[46], [47], [48]], and statistical analysis [49], 3) the classification, which uses extracted features to categorize the sounds as belonging to different disease categories. Popular classifiers include K-nearest Neighbors [[50], [51], [52], [53], [54]], Support Vector Machines [[55], [56], [57], [58], [59]], Gaussian Mixture models [60,61], and Artificial Neural Networks [36,55]. Fig. 4 visually illustrates this workflow, from preprocessing to classification.

### 3.1 The commonly considered dataset in the literature

Analyzing respiratory sound signals for lung disease diagnosis relies heavily on the quality and characteristics of available datasets. This section provides an overview of commonly used respiratory sound datasets, highlighting their features and applications in developing models that differentiate healthy from unhealthy lungs based on abnormal sound patterns. These details can be listed in Table 1, while some other datasets with their details are listed in Table 2.

**Table 1:** Classification of the common dataset and their description associated with the references that are used.

Dataset	Description	Used by Ref.
Respiratory Sounds Dataset ICBHI (RSD) 2017 [69]	Contains normal audio signals and three types of adventitious sounds: wheezes, crackles, and combined wheezes/crackles.	[30,48,[63], [64], [65], [66], [67], [68]]
HF_Lung_V1 [21]	Includes 9765 lung sound audio files (15 s each), labels for exhalation, inhalation, and irregular and regular adventitious sounds (rhonchus, wheeze, stridor).	[21]
Respiratory-Database@TR [71]	Short recordings, 12-channel lung sounds for each patient, multi-channel analysis, 5 COPD severity levels.	[70]
Own Generated Database [72]	Lung sounds were recorded with an e-stethoscope and a laptop.	[72]
Own Generated Database [73]	Two types: complete patient set (normal/abnormal) and sub-interval set (all patient measurements). Approx. 255 h of recordings.	[73]
Own Generated Database [74]	28 patient records with RSs non-stationary data collection. Separate training/testing sets with two recordings from different patients per class (except crackles/wheezes: 6 patients each). Sampling frequency: 44.1 kHz.	[74]
R.A.L.E. Repository [76]	Collection of digital recordings of healthy and diseased respiratory sounds.	[75]
R.A.L.E. Lung Sounds 3.0 [78]	Five normal breathing soundtracks, four crackle recordings, and four wheeze soundtracks. Band-restricted signal (7.5 Hz - 2.5 kHz).	[77]
Respiratory Sound Database [80]	920 recordings of varying durations from 126 patients. Annotations include the start/end timings of each respiratory cycle and the presence of wheeze/crackle.	[30,48,54,62,72,79]

**Table 2:** Lung sound datasets are considered with deep classifiers in the literature.

References	Dataset name	Samples	No. of classes
Zhao et al. [81], Rituerto-González et al. [82], Basu and Rana [2], Chanane and Bahoura [83]	ICBHI Challenge database	6898 cycles (5.5 h)	4 classes
Zhang et al. [84]	Munich-Passau Snore Sound Corpus (MPSSC)	828 snore events	219 subjects
Jeong et al. [85]	PASCAL Heart Sound Challenge (HSC) A and B datasets	A: 176 records B: 656 records	5 classes of heart sound
Jeong et al. [85], [86] Koike et al.	PhysioNet CinC Dataset	3240 audio files	2 classes (normal/abnormal)
Zheng et al. [87]	Gastrointestinal Sound Dataset	43,200 audio segments	6 kinds of body sound

Some notes were assigned with these tables; The R.A.L.E. Lung Sounds website serves as a valuable resource for anyone interested in respiratory health, offering a comprehensive collection of recorded lung sounds, and case studies. Datasets vary in size, recording duration, and types of sounds included. Some datasets are publicly available, while others require permission to access. This information is intended to help researchers choose the appropriate dataset for their lung disease diagnosis studies.

### 3.2 Feature extraction of lung sound signals

The choice of features utilized affects a model's performance in terms of how well or poorly it works. Therefore, it is crucial to take into account the numerous cutting-edge feature extraction techniques used in earlier studies. The usage of handcrafted characteristics diminishes when audio signals are represented generically, according to certain findings of earlier research [88]. The sound signal features can be divided into three classes.

• **Low-Level Features**: These characteristics include the rate of zero-crossing, energy, the amplitude envelope, and others. Typically, these are the statistical characteristics that are taken from the sound signal.
• **Average-Level Features**: These characteristics include beat and pitch level attributes such as MFCCs, note fluctuation patterns, and note onsets.
• **High-Level Features**: These are also called global features and they provide information about the whole sound (algorithmic aggregate of features for the raw audio signals).

Some audio characteristics, known as instantaneous features, provide information on a time scale of 20–100 ms (small chunks of audio signals). Segment-level characteristics are some that provide data for a 2–20 s time period. The modification of the signals to remove undesired noise and balance the time-frequency ranges requires the extraction of audio features from the time and frequency domains. The immediate information about the audio signals, such as the amplitude envelope, zero-crossing rate, and, energy of the signal, is obtained through the time domain-based feature extraction. The frequency content of the band energy ratio, audio signals, and other information is revealed through frequency domain-based audio feature extraction. To determine how quickly the spectral bands of an audio signal change, the time-frequency representation may be taken into account.

Once the audio features have been retrieved, an intelligent audio system can be created using either Deep Learning (DL) methods or more conventional ML algorithms. Automatic sound synthesis, classification, and composition are all done using intelligent audio systems. Deep learning architectures are made to classify/make symphonies, acting as a virtual composer, and to generate music, acting as a music classifier. The fact that the DL algorithms offer unstructured data distinguishes them from conventional ML algorithms. As a result, the DL has the ability to pass either the signal's spectrogram or the entirety of the raw audio signals as a collection of features.

Previous researchers have employed spectrogram properties extensively in a variety of sound categorization domains, such as using heartbeat sounds to identify cardiac conditions [86]. The MFCC's characteristics have demonstrated success in portraying sounds for the identification of respiratory illnesses [2].

In order to detect respiratory disorders using lung sounds, Ramesh et al. [89] suggested a combination of various feature extraction approaches. The feature extraction methods proposed include the following examples: MFCC, roll-off, flux, entropy, spread, spectral centroid, energy entropy, energy, and ZCR, which are some of the terms used to describe spectral properties. Similar investigations were conducted in Ref. [83] employing the mel-STFT, STFT, CQT, and lastly the combination of empirical mode decomposition (EMD) as four feature extraction approaches to characterize lung sound. The authors in Ref. [90] proposed an aggregated feature extraction method that combines local and global acoustic features.

According to our research, feature representation is essential to enhancing learning algorithms' efficiency in sound classification assignments. High dimensionality is typical of audio transmissions. A crucial step towards enhanced sound recognition accuracy is the development of efficient feature recognition methodologies, which can be achieved through the utilization of more effective feature representation approaches. Table 3 provides an overview of the feature extraction of audio-based lung disease recognition by deep learning algorithms.

**Table 3:** Feature extraction summary on audio-based lung disease recognition by deep learning algorithms.

Ref.	Feature extraction technique	Application
[[91], [92], [93]]	Mel Frequency Cepstral Coefficient (MFCC)	Respiratory diagnosis
[30,36,55,93,94]	Spectrogram	Heart and respiratory diagnosis
[30,38,55,94,95]	Short-time Fourier transform (STFT)	Medical sound classification
[96]	Mel-STFT	Lung disease recognition
[96]	Empirical mode decomposition (EMD)	Lung disease classification
[97]	Data de-noising autoencoder	Lung disease recognition
[55,83,93]	Constant Q transform	Lung and heart disease recognition
[84]	bag-of-Audio-word (BoAW)	Medical sound classification

**Fig. 5:**
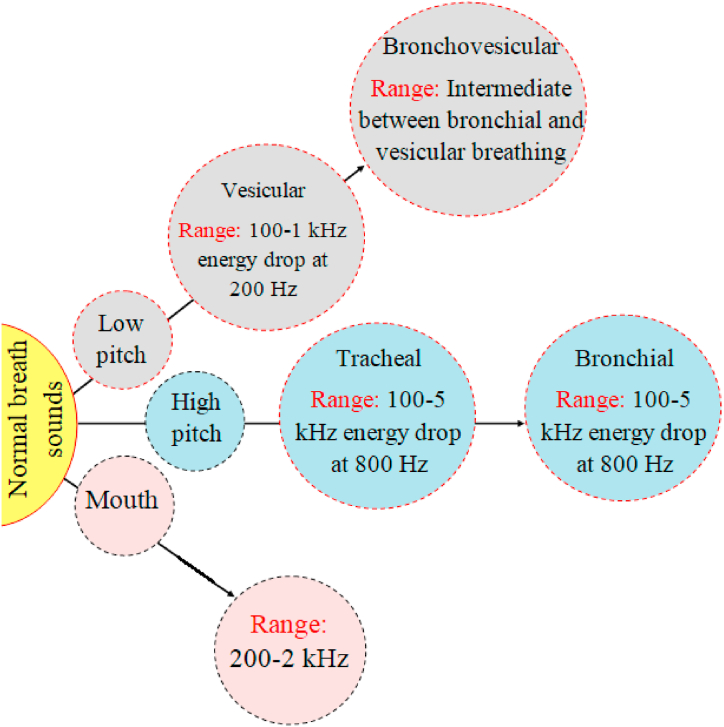
Lung normal sound features in terms of frequency and energy.

Lung disease alters the airflow within the chest, creating sound waves with unique characteristics. These characteristics, like the loudness (strength), tone (timbre or quality), and pitch (frequency) of the sound, can be analyzed to diagnose common lung disorders. Lung sound features such as frequency and power can be characterized as demonstrated in Fig. 5.

### 3.3 Pre-processing of lung sound signals

**Fig. 6:**
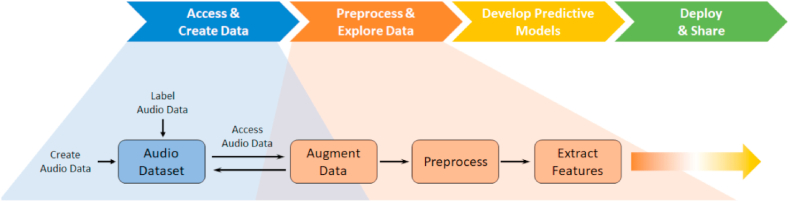
The steps for preparing the audio input data for deep learning.

Preprocessing audio files contains functions like trimming the audio to a consistent duration, removing regions of silence, and resampling audio files to a consistent sample rate. One of the well-known tools to perform these functions is the Signal Processing and DSP tools of MATLAB [98]. Audio includes unnecessary and frequently redundant information with highly dimensional data. Traditionally, low-level and MEL-frequency cepstral coefficients (MFCC) features, such as the spectral shape descriptors and zero-crossing rate, have been the dominant features resulting from sound waveforms as input data for deep learning architectures. Deep learning algorithms trained on these features are computationally efficient and often use less training input data. Advancements in deep learning models, large and well-labeled data sets, and increased access to computing power have reduced the dependence on hand-designed features. Fig. 6 demonstrates the steps for preparing the audio input data for training and showing the preprocessing step.

The quantization range of the raw audio can be reduced using a pre-processing step, which will improve the effectiveness of the deep learning model's output. Several studies like [[99], [100], [101], [102], [103]] apply μ-law to reduce the potential values of every calculation. The coefficient μ-law can be represented by equation (1) [104]:

(1) $$f\left(x\right)=sgn\left(x\right)\frac{\ln\;\left(1+\mu \left|x\right|\right)}{\ln\;\left(1+\mu \right)}$$

where −1 < *x* < 1 and μ denotes the level number produced behind the conversion. Even while non-linear quantization techniques like -law have drawn a lot of interest lately, the majority of current articles employ a normalized high-resolution signal as their input [105]. At last, other applications consist of the input signal linear quantization [[106], [107], [108]] and different models for less and most significant bits [109].

**Fig. 7:**
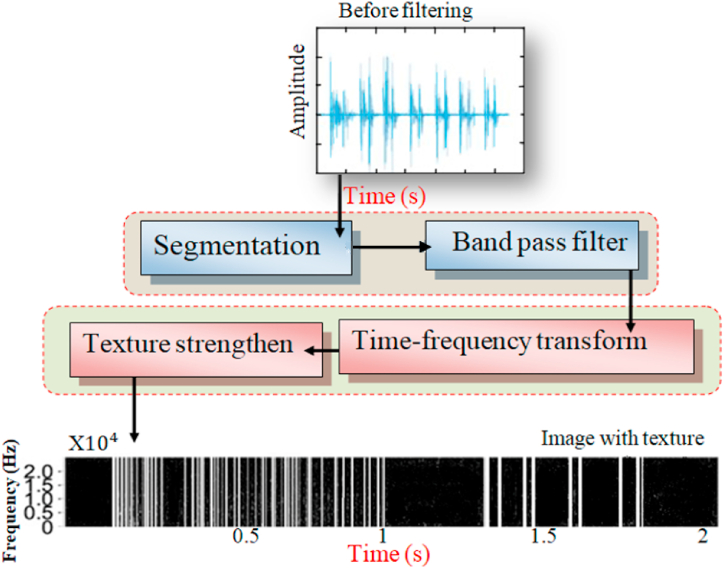
Block diagram of the signal preprocessing stage [110].

Sound recordings undergo several steps in the preprocessing stage. First, they are imported and re-sampled to a single rate (commonly 44,100 Hz) to ensure consistency across recordings made with different equipment. Next, each sound is cropped to a duration between 3 and 10 s by either padding shorter segments with zeros or trimming longer ones. Fig. 7 illustrates this preprocessing pipeline in a clear and concise manner.

### 3.4 Artifact removal methods

**Fig. 8:**
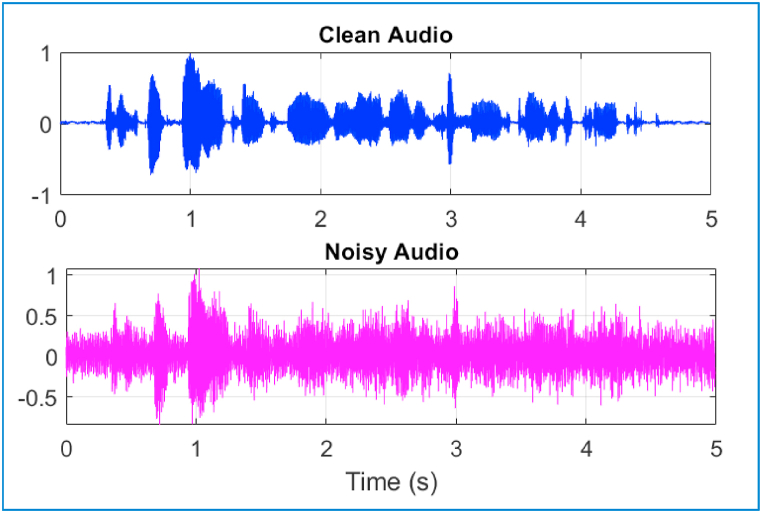
A clean audio signal (upper) and noise with air-conditioner sound (lower) with an 8 kHz sampling.

Audio artifact removal aims to subtract noise from the sound signals while improving the intelligibility and quality of the sound signal. Deep learning-based audio diagnosis techniques usually present artificial residual noises, particularly as the information phase is ignored in training targets [104], for example, the magnitude of the clean speech and its variations [111,112], or the ideal ratio mask [113,114]. This type of noise is typically extremely non-stationary, and in the middle-high frequency region, when audio power spectral density (PSD) is low, it nevertheless has a sizable power. A human listener will be able to detect and find the remaining noise PSD irritating when it reaches the noise masking threshold, according to the widely used human hearing model in wideband audio coding [115]. The residual noise issue has not yet been fully resolved, despite numerous significant efforts, including the use of sub-pixel convolution in densely connected neural networks (DCN) in Ref. [116] and the combination of multiple dilated CNN layers in gated residual networks with dilated convolutions (GRN) in Ref. [116] and recursive network with dynamic attention (DARCN) in Ref. [117]. An example of an audio waveform with an 8 kHz sampling frequency is shown in Fig. 8.

**Fig. 9:**
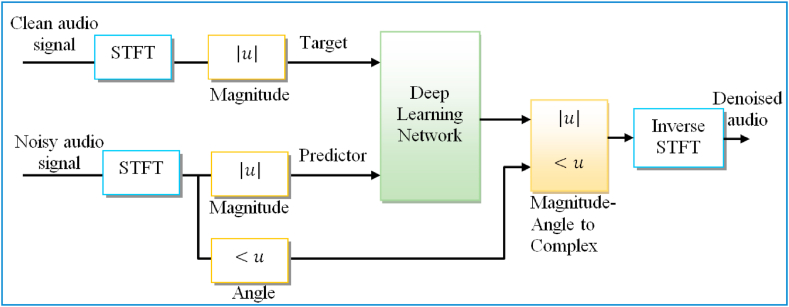
A fundamental diagram of the training process using a deep learning model.

Since the audio signal usually falls under 4 kHz, the first step is to down-sample the noisy and clean sound signals to 8 kHz to decrease the calculation weight of the model. The target and predictor network waveforms are the spectra magnitude of the clean and noisy sound signals, respectively. The architecture's output is the de-noised signal magnitude spectrum. To reduce the mean square error, the network regression of the predictor inputs is employed between the input target and its output. The de-noised sound signal is transformed to the time domain back by means of the phase of the noisy signal and the output magnitude spectrum [118]. The fundamental diagram of the training process for the deep learning model is demonstrated in Fig. 9.

**Fig. 10:**
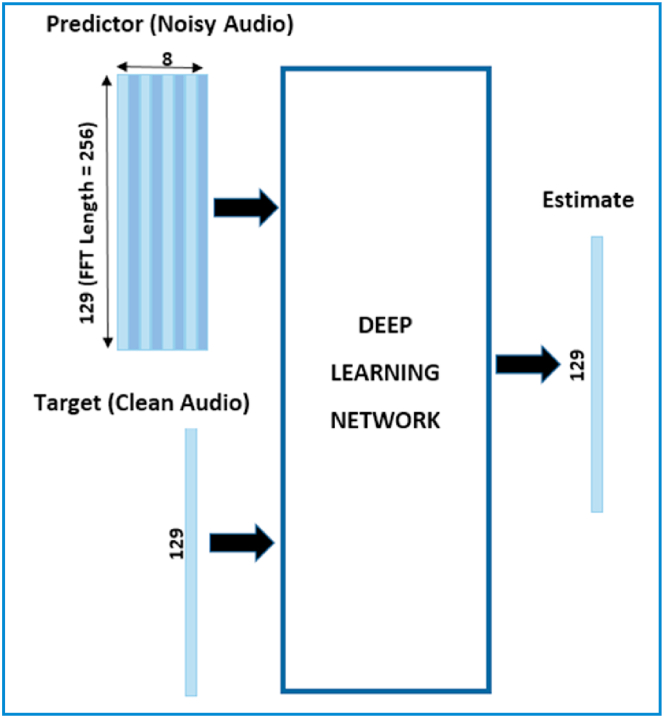
The estimated de-noising output is due to using a deep learning model from the clean and noisy audio of the predictor.

By using the Short-Time Fourier transform (STFT), the audio is transformed into the frequency domain. For the above audio signal, we use a Hamming window, an overlap of 75%, and a window length of 256 samples. Since the audio signal time-domain is real, there is no lead to any information losses. Therefore, the spectral vector size is reduced to 129 by reducing the negative frequencies corresponding to frequency samples. The input of the predictor contains 8 vectors of successive noisy STFT in such a way that all output STFT estimates are calculated according to the 7 previous noisy STFT vectors and the current noisy STFT as depicted in Fig. 10.

### 3.5 Lung-heart sound separation

**Fig. 11:**
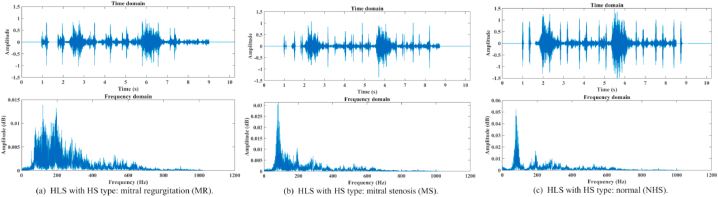
Samples of lung-heart sound in time and frequency domain for, unlike kinds of Heart sound.

Diagnoses of respiratory and cardiovascular conditions are frequently made using auscultation. Lung sound (LS) and Heart sound (HS) are both present in the lung-heart sound (LHS) produced by the stethoscope. Distinguishing LS and HS from the mixed signal is the first and most important step. Samples of lung-heart sound in time and frequency domain for unlike kinds of Heart sound are shown in Fig. 11 with a) mitral regurgitation, b) mitral stenosis, and c) normal.

**Fig. 12:**
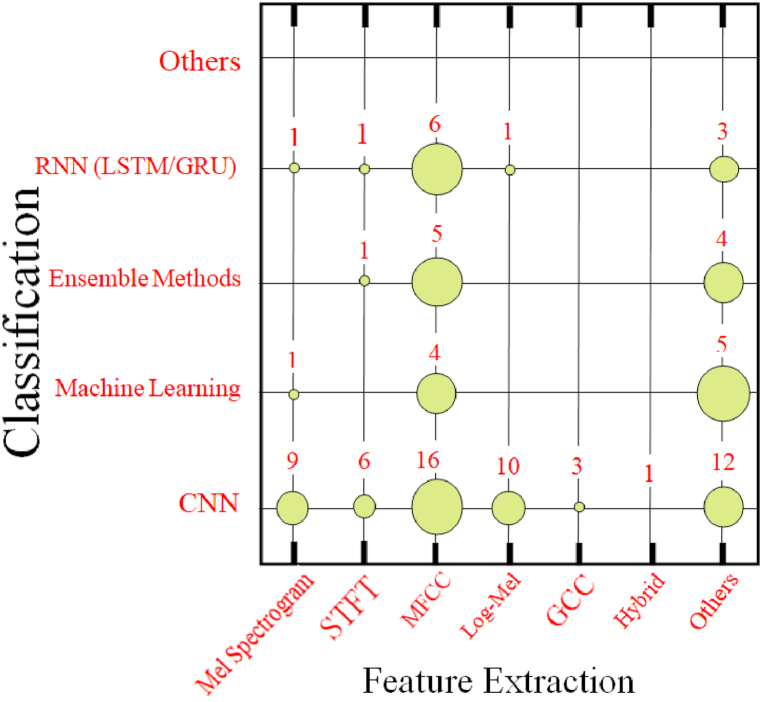
Graphical representation showing the number of publications in terms of circles with different sizes for crossing the classification with feature extraction methods.

LS and HS are essential diagnostic tools for cardiopulmonary diseases, providing valuable information about cardiovascular and respiratory health [[119], [120], [121]]. However, when doctors listen to the chest with a stethoscope (auscultation), LS and HS heavily overlap in both frequency and time, making it difficult to separate the mixed signal (LHS) using traditional BSS techniques [[122], [123], [124]]. Additionally, the variability of HS depending on the location and the patient's condition, coupled with the differing energy distribution across different types of HS, can lead to diverse characteristics of LHS [125]. This underscores the importance of researching BSS methods for LHS separation, as it has the potential to improve the quality of auscultation and aid physicians in diagnosis. Numerous researchers have actively explored and developed various BSS methods for separating LHS [[126], [127], [128], [129], [130]]. Table 4 lists a summary of the LHS separation techniques and their marked limitations.

**Table 4:** Summary of the LHS separation methods and their marked limitations.

Source	Method	Remarked limitation
Weibo et al. [131]	single-channel LHS partitioning not including a priori circumstances based on deep learning and nonnegative matrix factorization (NMF)	Effective but with complexity computations
[[132], [133], [134]]	Independent component analysis (ICA) for separating multivariate signals into additive subcomponents	ICA-based methods lack the ability to separate combined lung-heart sounds (LHS) recorded using a single sensor. This option maintains the original technical language and emphasizes the limitation of ICA for single-channel recordings.
Makkiabadi et al. [135]	convolutional BSS method using an orthogonal model	These methods rely heavily on pre-existing assumptions, which significantly limit their applicability.
Takada et al. [136]	higher-order statistics (HOS) method	
[137,138]	optimality criterion in signal processing using an Adaptive signal processing (ASP)	
Nersisson et al. [139]	Modified least mean square algorithm derived from an adaptive noise offset method	Methods based on adaptive signal processing (ASP) require a reference signal to separate lung-heart sounds (HLS). In practice, obtaining this reference signal can be challenging or even impossible.
Al-Naggar et al. [140]	A normalized last-mean-square with an adaptive noise termination algorithm	
Shah et al. [141]	Two unsupervised clustering with NMF-based HLS separation methods	Despite its ability to perform unsupervised learning and separate single-channel HLS without preconditions, NMF suffers from incomplete separation due to significant time-domain and frequency-domain overlaps arising from the inherent mathematical properties of matrix decomposition. While DL techniques offer ideal capabilities for feature extraction during HLS separation, the diverse nature of HLS signals can undermine the reliability of neural networks.
Lin et al. [142]	NMF-based constant Q transform for dimensionality reduction	
Shah et al. [143]	An unsupervised BSS and enhanced NMF with shared factors and method that does not require any training data	
Canadas et al. [144]	A spectrotemporal clustering by NMF	
Montoro et al. [145]	A parallel source partitioning system derived from NMF	
Sathesh et al. [146]	HS real-time signals from LS signals with ANN	
Tsai et al. [26]	A periodical deep autoencoder encoded method	

### 3.6 Deep learning methods

Fig. 12 presents a graphical depiction of the distribution of publications categorized by classification and feature extraction methodologies. The figure reveals that the majority of research papers employed convolutional neural networks (CNNs) for classification and Mel-frequency cepstral coefficients (MFCCs) for feature extraction. MFCC was also commonly used with Recurrent Neural Networks (RNNs), machine learning, and ensemble learning, as well as other feature-based methods that have been infrequently used with machine learning and ensemble methods. The significant sound-based classification deep learning algorithms used in this study are listed in Table 5.

**Table 5:** Sound-based classification deep learning algorithms.

Application	References	Categorization Method
Sound event detection	Ykhlef et al. [147]	Adaboost
Environmental acoustic scene and sound categorization	[[148], [149], [150]]	Audio Event Recognition Network (AReN)
Sound event recognition	Greco et al. [151]	Audio Event Recognition Network (AReN)
Acoustic scene classification	Ma et al. [152], Novotny et al. [153]	Autoencoder DNN
Environmental sound classification	Wyatt et al. [154]	Bidirectional Encoder Representations from Transformers (BERT)
Sound event detection, speaker recognition	Zhao et al. [81], Zheng et al. [87]	Bidirectional Gated Recurrent Neural Networks (BiGRU)
Various applications	[83,[85], [86], [87],^97^,^147^,[155], [156], [157], [158], [159], [160], [161], [162], [163], [164], [165]]	Convolutional Neural Network (CNN)
Various applications	[[166], [167], [168]]	Deep CNN (DCNN)
Various applications	[158,[169], [170], [171], [172]]	Deep neural network (DNN)
Sound classification	Ozer et al. [173]	DNN trained with Restricted Boltzmann Machine
Respiratory sound classification, speech emotion recognition, snore sound classification	[2,84,158]	Gated Recurrent Unit (GRU)
Acoustic scene classification, environmental sound classification	[148,150,174,175]	ResNet

### 3.7 Wavelet transform

**Fig. 13:**
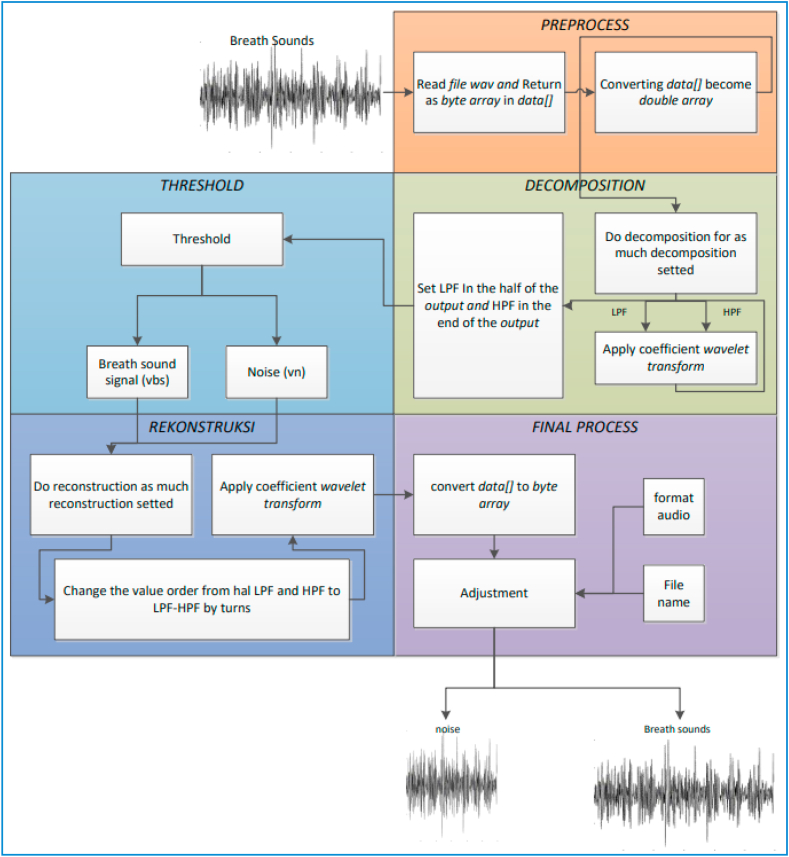
The Wavelet transforms as an element of the filtering or de-noising process [178].

Audio classification is the process of analyzing audio signals and assigning them to specific categories, such as identifying different types of musical instruments, classifying environmental sounds like bird songs or car horns, or even diagnosing medical conditions based on lung sounds [176]. Wavelet transform offers several advantages over other techniques for audio classification including: 1) Multi-resolution analysis, where the wavelets can capture both high-frequency and low-frequency components of the audio, providing a more comprehensive understanding of the signal compared to Fourier Transform (FT), which primarily focuses on frequency analysis. 2) Time-frequency localization, where the wavelets analyze the signal in both time and frequency domains, allowing us to identify specific events and their location within the audio. This is crucial for classifying sounds with transient features, like percussive instruments or coughs. 4) Robustness to noise due to that the wavelet functions are less sensitive to noise compared to Fourier basis functions, making them well-suited for analyzing noisy audio recordings [177]. Using the WPT, the frequency sub-bands of lung sound waves were divided into statistical characteristics that described the distribution of wavelet coefficients. An ANN is used to categorize lung sounds as normal, wheezing, or crackling. A microcontroller with this classifier's programming was used to create a portable, automated system for analyzing and diagnosing respiratory function. A method for differentiating between two kinds of lung sounds was offered in the study [177]. The foundation of the suggested method was a study of wavelet packet decomposition (WPD). From a range of patients, information on normal, abnormal, and normal lung sounds was gathered. Expiration and inspiration were separated into separate portions for each signal. They created compressed and significant energy characteristic vectors using their multi-dimension WPD factors, which they then fed into a CNN to identify lung sound features. Despite having great identification efficiency as shown by numerous exploratory outcomes, this characteristic extraction approach is not yet ready for clinical usage. As seen in Fig. 13, the Wavelet transform plays a part in the filtering or de-noising process.

The general steps involved in using Wavelet transform for audio classification.

• Preprocessing: The audio signal is preprocessed by removing noise, normalizing amplitude, and segmentation into smaller frames.
• Feature extraction: Wavelet transform is applied to each frame to extract features like coefficients, energy distribution, and signal entropy.
• Feature selection: Relevant features are selected based on their ability to discriminate between different classes.
• Machine learning: The extracted features are utilized as input for a variety of machine learning algorithms, including Support Vector Machines (SVMs), Random Forest, and Neural Networks, in order to train a classification model.
• Classification: The trained model is used to classify new audio signals based on their extracted features.

The benefits of using the wavelet technique include 1) high accuracy in identifying various audio classes, 2) robustness to noise and environmental variations, and 3) effectiveness for analyzing transient and non-stationary signals. In contrast, the challenges when using such a method with audio signals include that it requires careful selection and tuning of wavelet functions, computational complexity can be high, and feature selection may require domain expertise.

Wavelets constitute a family of functions $\varphi_{a,b}t$ derived through the process of translation and dilation applied to a single function designated as the “mother wavelet" φ(t) [64] as described in equation (2):

(2) $$\varphi_{a,n}t=\frac{1}{\sqrt{a}}\varphi \;\left(\frac{t-n}{a}\right),a>0,n\in R$$

The function “mother wavelet" is used to examine signals by scaling and shifting its shape to match the desired frequency and time characteristics of the signal. The resulting discrete wavelet transform can be expressed mathematically as shown in equation (3):

(3) $$\varphi_{j,k}\left(t\right)=2^{\frac{j}{2}}\varphi \left(2^{j}t-k\right)$$

Equation (4) represents the dimensionless signal-to-noise ratio, which measures the power of a signal relative to background noise during recording [65].:

(4) $$SNR=\frac{P_{signal}}{P_{noise}}={\left(\frac{A_{signal}}{A_{noise}}\right)}^{2}$$

where $A_{signal}$ and $A_{noise}$ represents the root mean square (RMS) of signal amplitude, and noise amplitude, respectively. $P_{\text{signal}}$ is the mean of signal power, and $P_{noise}$ is the mean of noise power [179,180].

Therefore, Wavelet transform is a powerful tool for audio classification, offering a rich representation of the signal and capturing both spectral and temporal information. Its ability to analyze both high-frequency and low-frequency components makes it suitable for classifying a wide range of audio signals. However, it is crucial to carefully select the wavelet functions and features to achieve optimal performance. With the increasing power of computational resources and advancements in machine learning algorithms, wavelet transform is expected to play an even more significant role in audio classification tasks in the future.

## 4 . studies evaluate lung screening

Several studies discussed lung nodule analysis and screening with deep learning in respiratory disease diagnosis. Studies [[181], [182], [183]] reviewed progressive deep learning algorithms for screening and respiratory nodule analysis, emphasizing their applications in medical settings. The paper [181] compared the performance of different networks for lung nodule detection, highlighting their limitations and potential future directions. The study [182] discussed the relevance of molecular and cellular processes in lung disease diagnosis. The study [183] reported promising results from small-scale studies using deep learning convolutional neural networks (DLCNNs) for various diagnostic procedures, including forced oscillation tests, lung sound analysis, breath, and telemedicine analysis.

Lung Cancer diagnosis through medical image analysis was discussed by the papers [[184], [185], [186], [187], [188], [189]] that reviewed the use of medical image analysis for lung cancer diagnosis, while the study [186] highlighted the importance of this research by emphasizing the high global mortality rate of lung cancer, with 1.76 million deaths reported in 2018. The paper [187] further emphasized that lung cancer exhibits the highest incidence rate of cancer mortality across both genders, accounting for more than one-fourth of all global cancer fatalities. The potential of deep-learning CNNs for lung disease assessment is being actively investigated, with numerous studies contributing novel and effective methods [190]. To showcase the significance of these publications, Table 6 summarizes the analyzed samples, the CNN algorithm types tested on image or sound data, and their key findings.

**Table 6:** Summarizing the analyzed samples, the CNN algorithm types tested on image or sound data, and their key findings.

Algorithm	Performance				Splitting Strategy	Ref.
	Specificity	Sensitivity	Accuracy	Score		
VGG16	–	–	63.09%	–	10-fold CV	[191]
SVM	–	–	49.86%	–	official 60/40	[192]
GMM Classifier	90%	90%	85.00%	–	–	[193]
–	0.56	–	86% Wheeze	–	–	[194]
CNN	–	–	74.3%	–	random 70/30	[195]
ResNet + SE + SA	81.25%	17.84%	–	49.55%	official 60/40	[196]
ANN	86%	86%	76.00%	–	–	[91]
bi-ResNet	69.20% 80.06%	31.12% 58.54%	52.79% 67.44%	50.16% 69.30%	official 60/40 random 10-fold CV	[197]
CNN-MoE	68% 90%	26% 68%	–	47% 97%	official 60/40 random 5-fold CV	[28]
CNN	72.3% 83.3%	40.1% 53.7%	–	56.2% 68.5%	official 60/40 interpatient 80/20	[198]
CNN	–	–	81.62%	–	random 75/25	[199]
ResNet	79.34% 82.46%	47.37% 37.24%	– 73.69%	58.29% 64.92%	official 60/40	[200]
CNN-RNN	84.14%	48.63%	–	66.38%	interpatient 80/20	[201]
Deep Belief Networks (DBN)	93.66% 73.34%	93.33% 67.23%	95.83% 70.278%		–	[202]
RNN	73%	58.4%	–	65.7%	interpatient 5-fold CV	[203]
CNN	81%	28%	–	54%	official 60/40	[204]
CNN-LSTM with FL	84.26% –	52.78% 60.29%	76.39% 74.57%	68.52% –	Interpatient 10-fold CV LOOCV	[30]
HMM SVM	56.69% 77.80%	42.32% 48.90%	49.50% 49.98%	39.37% 49.86%	official 60/40	[205]
ANN	100%	97.8%	98.3%	–	–	[74]
HMM	–	–	–	39.56%	official 60/40	[206]
GMM	92.8%	43.7%	80.00%	–	–	[75]
RBF SVM Classifier	86.56 (±0.36)	86.83 (±0.43)	86.71%	–	–	[73]
ResNet + NL	63.20% 64.73%	41.32% 63.69%	–	64.21% 52.26%	official 60/40 interpatient 5-fold CV	[207]
CNN	–	–	94.24%	93.6%	–	[72]
SVM	83.86%	82.06%	87.18%	82.67%	–	[77]
CNN	–	–	95.56%	–	–	[7]

Table 6 highlights several published articles and their achievements in identifying consolidation in Pediatric Chest X-rays. Studies [[208], [209], [210], [211]] developed a novel problem-based architecture capable of analyzing chest X-rays. This architecture incorporates a three-step pre-processing stage to enhance model generalizability.

For verifying the reliability of model outputs, an occlusion test is applied to visualize the predicted areas and confirm their relevance. To ensure the model's universality, the researchers validated its performance on a separate dataset. Their findings suggest that the developed models hold promising potential as computer-aided diagnosis tools in clinical practice. Additionally, the authors conducted a comprehensive analysis of existing datasets and prior studies. Their analysis revealed that neglecting certain precautions could lead to misleading results.

The greatest outcomes in the field of sound classification now come from a variety of convolutional deep learning techniques with memory, such as LSTM, RNN, and their variants like bidirectional gated recurrent neural networks (BiGRU) and time-delay neural network (TDNN). Deep learning models frequently need input data that is interpreted as images and organized as matrices. Mel frequency cepstral coefficients and MEL spectrograms are ideal feature extraction techniques that can be utilized in this situation to turn single-dimensional sound sequences into visuals that can be utilized to train deep learning models. Additionally, choosing the optimum representation of sound data is essential for enhancing performance outcomes in sound classification, in addition to including an efficient data augmentation technique.

## 5 Existing literature gaps

Several challenges hinder researchers in acoustic signal analysis and identification of respiratory diseases.

1. Noise: Respiratory acoustic samples often contain significant noise, making analysis and identification more difficult [212].
2. Imbalanced Data: The distribution of respiratory audio samples often varies across different diseases [92]. This leads to imbalanced datasets, requiring careful balancing techniques to avoid overfitting and ensure accurate predictions.
3. Computation Cost: Traditional non–CNN–based deep learning approaches often require complex architectures with high computational demands [213]. This limits their practical applicability due to the need for expensive high-performance computing resources. Training models on such architectures can be extremely time-consuming, even with sufficient resources. This problem is further exacerbated by the time-intensive nature of traditional physical identification methods by physicians, which often require multiple hospital visits and prolonged wait times.
4. Feature Extraction: Effective feature extraction strategies are crucial for developing successful deep learning models, especially when aiming for high accuracy. Optimal feature selection helps reduce data redundancy and improve model efficiency throughout the analysis process.
5. Algorithm Choice: Selecting the appropriate algorithm is essential for achieving the desired results. Researchers should experiment with various algorithms to identify the one that best aligns with the specific objectives of the study.
6. Dataset Selection: Obtaining and maintaining high-quality, noise-free datasets is critical for building robust and reliable deep learning models. Proper preprocessing of training data is essential to ensure accurate and generalizable results.

## 6 Conclusions

This paper significantly advances the field of sound-based lung diagnosis by providing a comprehensive and critical analysis of the current landscape of machine and deep learning techniques, offering a valuable resource for researchers and practitioners. This work showcases the shortcomings and influence of machine learning strategies by determining the areas and obstacles of improvement that would benefit substantially prospective study concentrating on the necessity to improve sound-based deep learning classification and enhance lung diagnosis methods for large datasets.

Deep neural network applications in medicine were thoroughly examined in a number of research publications. As a result of various selection approaches, more than 180 research publications were selected, with more than 120 of them being presented in further depth. Overall, deep learning techniques based on sound have shown promise in the identification of lung illness. On the other hand, much research is still required to support current findings and broaden the medical community's acceptance of them. This information should be useful to medical professionals, researchers using sound-based deep learning, and companies that manufacture this technology.

## Data availability statement

Data will be made available when requested.

## CRediT authorship contribution statement

**Ahmad H. Sabry:** Writing – original draft, Writing – review & editing, Validation, Project administration, Investigation, Data curation, Conceptualization. **Omar I. Dallal Bashi:** Writing – review & editing, Software, Methodology, Data curation, Conceptualization. **N.H. Nik Ali:** Writing – review & editing, Validation, Supervision, Funding acquisition, Formal analysis. **Yasir Mahmood Al Kubaisi:** Writing – review & editing, Validation, Software, Resources, Project administration.

## Declaration of competing interest

The authors declare that they have no known competing financial interests or personal relationships that could have appeared to influence the work reported in this paper.
